# Loneliness in the workplace: a mixed-method systematic review and meta-analysis

**DOI:** 10.1093/occmed/kqad138

**Published:** 2024-01-29

**Authors:** B T Bryan, G Andrews, K N Thompson, P Qualter, T Matthews, L Arseneault

**Affiliations:** Social, Genetic and Developmental Psychiatry Centre, Institute of Psychiatry, Psychology and Neuroscience, King’s College London, London, UK; Social, Genetic and Developmental Psychiatry Centre, Institute of Psychiatry, Psychology and Neuroscience, King’s College London, London, UK; School of Psychology, Cardiff University, Cardiff, UK; Social, Genetic and Developmental Psychiatry Centre, Institute of Psychiatry, Psychology and Neuroscience, King’s College London, London, UK; Manchester Institute of Education, University of Manchester, Manchester, UK; Social, Genetic and Developmental Psychiatry Centre, Institute of Psychiatry, Psychology and Neuroscience, King’s College London, London, UK; School of Human Sciences, University of Greenwich, London, UK; Social, Genetic and Developmental Psychiatry Centre, Institute of Psychiatry, Psychology and Neuroscience, King’s College London, London, UK

## Abstract

**Background:**

Loneliness is a risk factor for a range of mental and physical health problems and has gained increasing interest from policy-makers and researchers in recent years. However, little attention has been paid to loneliness at work and its implications for workers and employers.

**Aims:**

Identify workplace, health and personal factors associated with workplace loneliness.

**Methods:**

We searched five databases (PubMed, MEDLINE, EMBASE, PsycINFO and EBSCO Business Source Complete) for relevant articles published from 1 January 2000 to 23 February 2023. Quantitative data were synthesized using narrative synthesis and random-effects meta-analysis of correlation coefficients. Qualitative data were synthesized using thematic synthesis. Evidence quality was appraised using the Mixed-Methods Appraisal Tool.

**Results:**

We identified 49 articles meeting the inclusion criteria. Pooled results indicate that workplace loneliness was associated with lower job performance (*r* = −0.35, 95% CI −0.49, −0.21), reduced job satisfaction (*r *= −0.34, 95% CI −0.44, −0.24), worse worker–manager relationship (*r* = −0.31, 95% CI −0.38, −0.24) and elevated burnout (*r* = 0.39, 95% CI 0.25, 0.51). Qualitative results suggest links between loneliness and inadequate workplace social interactions and mental health problems. As most studies used cross-sectional data and few adjusted for potential confounders, the direction and robustness of the associations remain untested.

**Conclusions:**

Our results indicate that loneliness is associated with poor occupational functioning and well-being among workers. Results also show that loneliness is associated with modifiable aspects of the work environment, suggesting that the workplace may offer a fruitful avenue for interventions targeting loneliness.

## Teaser

While loneliness has been identified as a risk factor for poor health, experiences of loneliness at work are not well understood. This review was conducted to identify workplace, health and personal factors associated with workplace loneliness. We found evidence that workplace loneliness is associated with poor occupational functioning and well-being among workers, as well as modifiable aspects of the work environment. Longitudinal research is needed to understand the direction of these associations.

**Key learning points**

**What is already known about this subject:**

- While loneliness has been identified as a risk factor for a range of physical and mental health problems, little attention has been paid to experiences of loneliness at work.
- The risk factors and consequences of workplace loneliness are unclear.
- No review has examined associations between workplace loneliness and the work environment or health and well-being.

**What this study adds:**

- Meta-analyses indicate that loneliness is associated with higher burnout symptoms, lower job performance and reduced job satisfaction.
- Quantitative and qualitative evidence suggest loneliness is also associated with modifiable aspects of the work environment, particularly the worker–manager relationship.
- This study highlights the lack of longitudinal data and adjustment for correlates for testing the direction and robustness of these associations.

**What impact this may have on practice or policy:**

- Our results indicate that loneliness is associated with reduced well-being and occupational functioning among workers, which may have costs for employers.
- Employers and practitioners should consider loneliness among other aspects of workers’ well-being.
- Interventions targeting managers’ behaviour may be effective in addressing loneliness among workers.

## Introduction

Workplace loneliness has received increased public and policy attention in the wake of the coronavirus disease 2019 (COVID-19) pandemic. Rapid changes to working patterns have led to heightened concerns about the impact of work-related loneliness on health and well-being [1], with a recent report from the UK All Party Parliamentary Group on Tackling Loneliness and Connected Communities calling for employers to tackle loneliness within their organizations [2]. These experiences of working during the pandemic have highlighted the longstanding issue of loneliness, with the New Economics Foundation estimating in 2017 that loneliness costs UK employers £2.5 billion per year [3].

Loneliness is defined as subjective dissatisfaction arising from a mismatch between the quality and quantity of relationships a person desires and has in reality [4]. Loneliness is an insidious problem that is experienced by individuals across age groups [5], socio-economic strata and gender [6], with significant implications for health [7]. Loneliness is an important risk factor for poor mental and physical health outcomes, including depression, anxiety, cardiovascular disease [7] and mortality [8].

This association between loneliness and health is particularly pertinent in the workplace. Workplaces play an important role in adults’ lives, who spend a large amount of time and may develop meaningful relationships at work. Dissatisfaction with workplace relationships may engender loneliness, with implications for the emergence of mental health problems. In light of the link between loneliness and poor mental health, and the emergence of common mental disorders as the leading cause of sickness absence in high-income countries [9], loneliness may be an avenue for targeting workers’ health and well-being.

Workplace loneliness may also negatively impact workers’ occupational functioning, with implications for both workers and employers. Management researchers highlight the importance of relationships in organizations and have found that work-related motivation is lifted by social, as well as financial rewards [10], such that employees’ performance may be impacted when their social needs are unmet. Loneliness is also associated with difficulties entering the workforce, with lonely young people less optimistic about their careers [6]. Within this context, loneliness may have consequences for workers’ career progression, and productivity costs for employers and society.

While loneliness is a risk factor for poor health and socio-economic outcomes and has been studied widely among students and older people, few studies have examined loneliness among workers [1]. Workplace risk factors and the health and occupational consequences of work-related loneliness are not well established [11]. Further, there is disparate evidence on the topic given that workplaces are researched mostly by occupational health and management researchers, while the loneliness literature has emerged largely from psychology and medicine. A review of the literature is needed to connect this evidence and identify gaps that can be addressed by future research.

This mixed-methods review clarifies the evidence base around workplace loneliness. In particular, we address three questions: (1) are personal and workplace factors associated with increased work-related loneliness?, (2) is workplace loneliness associated with health and work-related difficulties?, and (3) does workplace loneliness measurement influence research findings?

## Methods

We conducted searches and study selection according to PRISMA 2020 guidelines [12]. All data, code and research materials are available at github.com/bridgetbryan/work-loneliness-review. This review was pre-registered on PROSPERO, CRD42021255553 crd.york.ac.uk/prospero/display_record.php?ID = CRD42021255553.

We searched five databases (PubMed, MEDLINE, EMBASE, PsycINFO and EBSCO Business Source Complete) to identify relevant articles. The search combined terms related to (1) loneliness, (2) the workplace, and (3) mental health, physical health, personal characteristics and work-related outcomes (full detail in Table 1, available as Supplementary data at *Occupational Medicine* Online). We also searched selected papers’ reference lists to identify relevant articles. Studies were eligible for inclusion if they met five criteria detailed in Table 1.

**Table 1.** Criteria for inclusion in systematic review

Domain	Inclusion criteria
Sample	Examines a sample of workers. Samples including individuals in formal employment, informal work or self-employment were eligible. Samples of student-workers, individuals on sick leave, or forced or child labour were excluded.
Phenomenon of interest	Presents original data on work-related loneliness. Quantitative studies that measured work-related loneliness and qualitative studies that named loneliness as a theme were eligible.
Correlates of interest	Analyses the association between work-related loneliness and workplace factors (such as job design), health or personal attributes (such as personality).
Study design	Quantitative methods including cross-sectional, cohort and case-control studies, as well as qualitative study designs.
Publication status and language	Published in English in peer-reviewed journals from 2000 to 23 February 2023.

After removing duplicates, we screened titles and abstracts to identify potentially relevant articles using Rayyan software [13]. We reviewed full-text versions of the retained articles, as well as studies identified in the reference list search, to determine whether they met inclusion criteria. Data were extracted using a custom-designed spreadsheet. The full list of variables extracted are detailed in Table 2.

**Table 2.** Study characteristics extracted

Study characteristic	Description
Author discipline	Discipline of study authors based on authors' departmental affiliation.
Year of publication	Year of print publication as indicated in the published article.
*N*	Sample size relating to loneliness data.
Proportion of women	Proportion of women participants.
Sample occupation	Information on the occupation of each sample.
Age (mean, SD)	Mean and standard deviation of the sample in years.
Age (range)	Minimum and maximum age of the sample in years.
Country	Country in which the study participants primarily worked.
Loneliness measure used	Information on the measure used to assess workplace loneliness in the sample. For standard instruments, this included the version of the scale and the number of items. For bespoke measures, this included the number of items and sample items.
Loneliness terminology	Information on how the experience of workplace loneliness was described (e.g. ‘work-related loneliness’, ‘occupational isolation’).
Methodology	Whether the study used a qualitative or quantitative method.
Method (data collection)	Information on how data was collected. For quantitative studies, this was often cross-sectional surveys. For qualitative studies, this was typically semi-structured interviews or focus groups.
Analysis strategy	Technique used to analyse the association between loneliness and other variables in the study.
Loneliness correlates	Non-loneliness variables analysed in relation to loneliness. Variables were grouped into workplace, health and personal factors.
Results—quantitative	Results of unadjusted and adjusted associations between workplace, health or personal factors and workplace loneliness.
Results—qualitative	Information about the key themes identified in the study, including the thematic structure, key quotes and interpretation.

The methodological quality of each article was assessed by two researchers (B.T.B. and G.A. or K.N.T.) independently using the Mixed-Methods Appraisal Tool [14]. Where discrepancies arose, we discussed differences and reached a consensus.

Quantitative data were summarized using random-effects meta-analysis of correlation coefficients, where at least four studies reported a bivariate association between work loneliness and a workplace, health or personal variable. Meta-analyses were performed in Stata 17 [15] using the *meta summarize* command. Where a correlate of workplace loneliness was analysed in too few studies to be meta-analysed, data were narratively synthesized in line with Synthesis Without Meta-Analysis guidelines (SWiM) [16]. Qualitative data were synthesized by two researchers (B.T.B. and G.A.) who applied Thomas and Harden’s thematic synthesis approach [17] using NVivo software [18]. Full details of the meta-analyses and thematic synthesis methods are provided in Supplement B (available as Supplementary data at *Occupational Medicine* Online).

## Results

**Figure 1.**
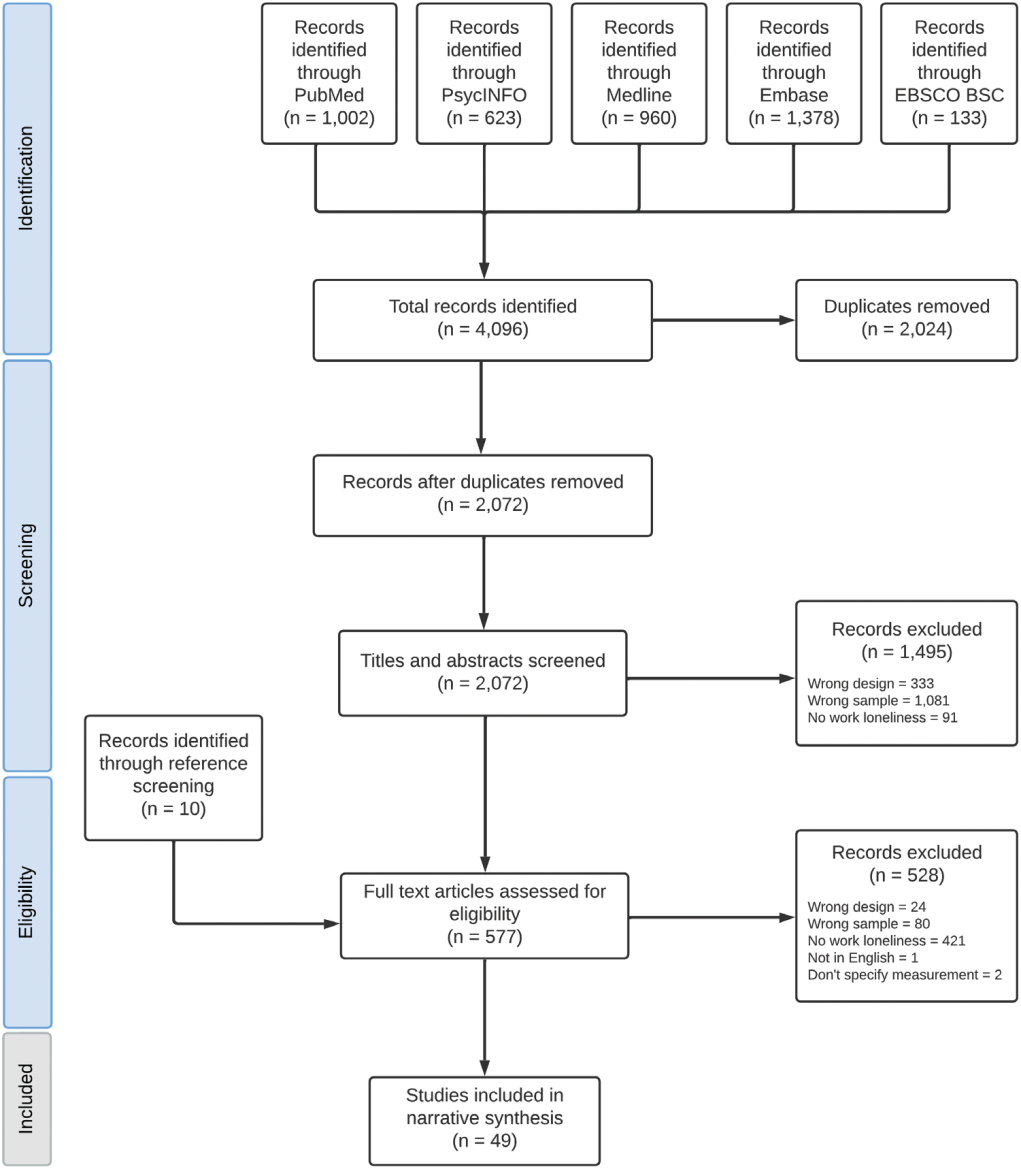
PRISMA flowchart of the screening and study selection process.

The search generated 4096 articles. After removing duplicates and screening titles and abstracts, we identified 567 potentially relevant articles (Figure 1). Ten additional articles were identified from reference list screening. We reviewed 577 full-text articles and found that 49 met inclusion criteria (full list of articles detailed in Supplement C). There were sufficient studies presenting associations between workplace loneliness and job performance, job satisfaction, leader-member exchange (LMX) and burnout to be pooled for meta-analysis.

The majority of articles in this review were published since 2015, with 43% published since 2020. The articles cover 14 271 workers across 23 countries. Over two-thirds of the articles included workers from a specific occupational group, such as healthcare workers or teachers; the remaining articles covered multiple occupations. The characteristics of the included studies are summarized in Table 3.

**Table 3.** Characteristics of studies included in systematic review

Study characteristics (*n* = 49)	*n*(%)
Year of publication	
2000–2004	2(4)
2005–2009	2(4)
2010–2014	5(10)
2015–2019	19(39)
2020–2022	21(43)
Author discipline	
Agricultural studies	1(2)
Business management	23(47)
Education	4(8)
Medicine, nursing, health, sport science	7(14)
Psychology, psychiatry	11(22)
Social science	3(6)
Methodology	
Quantitative	33(67)
Cross-sectional, time-lag or weekly diary study	28(57)
Longitudinal, experience sampling	5(10)
Qualitative	16(33)
Focus group	3(6)
Qualitative survey	2(4)
Semi-structured interview	10(20)
Semi-structured interview, blog analysis	1(2)
Sample size	
<100	17(35)
100–500	24(49)
500–1000	6(12)
>1000	2(4)
Geographical region	
Asia	6(12)
Australia/New Zealand	4(8)
Central/South America	1(2)
Europe	16(33)
Middle East	8(16)
Multiple continents	1(2)
North America	13(27)
Sample occupation	
Agricultural, horticultural workers	4(8)
Business owners	1(2)
Healthcare workers	8(16)
Knowledge workers or salespeople	9(18)
Manual workers	2(4)
Professional athletes	2(4)
Teachers	3(6)
Transport workers	2(4)
Multiple occupations	18(37)
Mean age in years	
<30	3(6)
30–39	15(31)
40–49	10(20)
50+	1(2)
Not specified	20(41)
% sample female (*n* = 44)	(48)

The studies varied in methodological quality. Several quantitative studies did not adjust for potential confounders in analyses. Many of the qualitative studies did not identify or justify the qualitative approach used or provided few quotes to support interpretation.

The quantitative studies in the review presented findings on the association between loneliness and workplace factors, health and well-being, and personal attributes.

**Figure 2.**
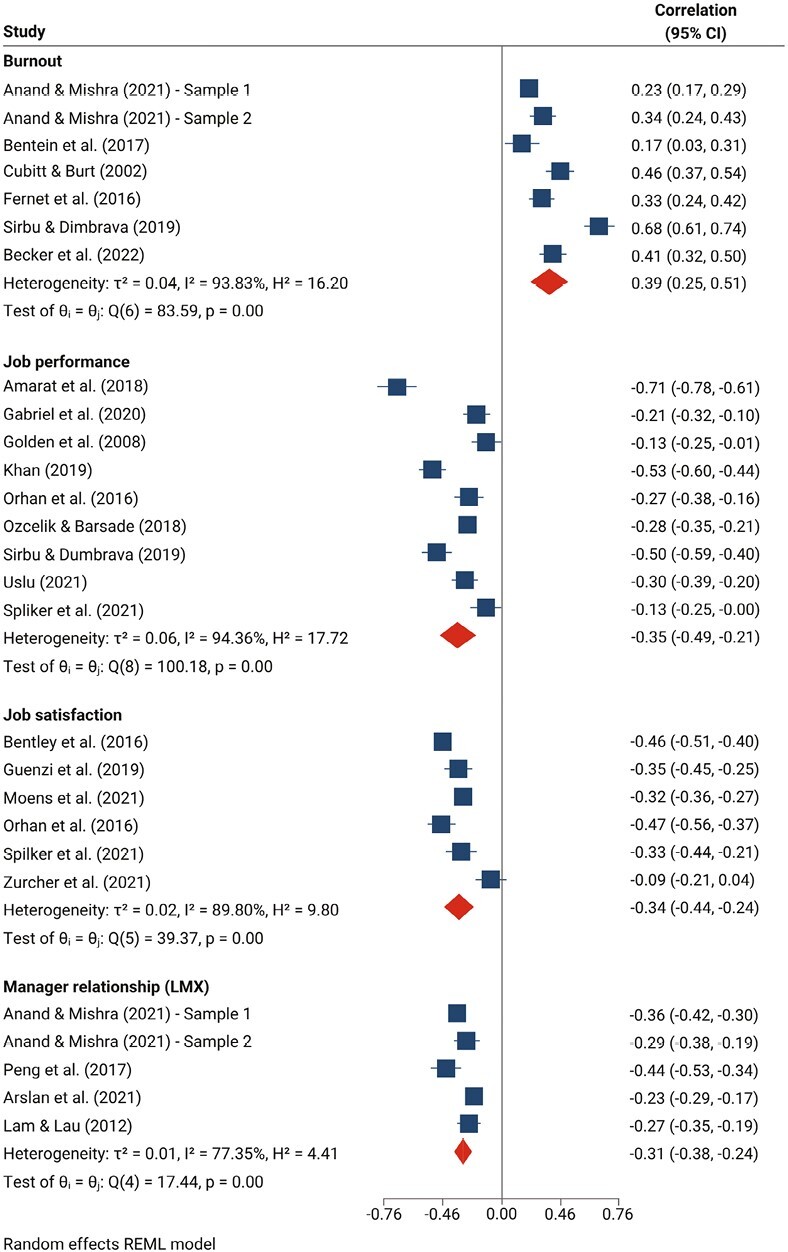
*Associations between workplace loneliness and burnout, job performance, job satisfaction and worker–manager relationship quality.* CI = confidence interval [19–30].

Among studies examining loneliness and workplace factors, the association between loneliness and workers’ attitudes and outcomes was the most widely studied in the literature. All nine studies that examined the association between workplace loneliness and job performance reported a negative association, with a moderate negative pooled correlation with considerable heterogeneity (*r* = −0.35, 95% confidence interval [CI] −0.49, −0.21, *I*^2^ = 94%) (Figure 2). We similarly found a moderate pooled correlation between workplace loneliness and lower job satisfaction (*r* = −0.34, 95% CI −0.44, −0.24, *I*^2^ = 90%).

Four studies examined loneliness and work engagement. Two studies found small cross-sectional correlations (*r* = −0.14, *P* < 0.05; *r* = −0.22, *P* < 0.01) [31,32], and one reported an association in both bivariate (*r* = −0.51, *P* < 0.01) and multivariate analyses (β = −0.36; *P* < 0.01) [33]. However, the association was not significant in multilevel path analyses [32]. Evidence on the association between loneliness and workers’ intention to leave their jobs was similarly mixed, with both negative (*r* = −0.28, *P* < 0.01) [34] and positive associations reported (*r* = 0.31, *P* < 0.01; *t* = 14.42, *P* < 0.01) [35,36]. Workplace loneliness was also associated with lower productivity (*r* = −0.16, *P* < 0.05; *r* = −0.42, *P* < 0.01) [33,37].

Studies also examined the link between loneliness and the workplace social environment. Four studies using five samples assessed the association between the quality of the relationship between a worker and their manager, operationalized using the LMX questionnaire [38]. These studies consistently reported a negative association between loneliness and lower relationship quality, with a small-to-moderate pooled correlation (*r* = −0.31, 95% CI −0.38, −0.24, *I*^2^ = 77%) (Figure 2). Beyond the worker–manager relationship, two papers found evidence for an association between loneliness and workplace social support (*r* = −0.29, *P* < 0.01; *r* = −0.49, *P* < 0.001) [39,40]. Evidence on the role of remote working was mixed. While two studies of workers during the COVID-19 pandemic found that the proportion of work conducted remotely was weakly associated with loneliness (*r* = 0.12, *P* < 0.01; *r* = 0.14, *P* < 0.05) [37,41], two papers published before 2020 found no association [34,40].

A smaller number of studies examined the link between job characteristics and loneliness. Two studies examined job autonomy and found mixed results with one study reporting a negative association (*r* = −0.23, *P* < 0.01) [42] and another finding no association [33]. Becker and colleagues [43] report a negative association between job control and loneliness (*r* = −0.27, *P* < 0.01).

In addition to workplace factors, 14 studies reported data on the association between work-related loneliness and mental health or well-being. Burnout was most frequently examined, with six studies assessing its association with loneliness across seven samples. All effect sizes were positive and significant (Figure 2), with a moderate significant pooled correlation between burnout and work-related loneliness (*r* = 0.39, 95% CI 0.25, 0.51). These results should be considered in light of substantial heterogeneity (*I*^2^ = 94%).

Four studies reported an association between work-related loneliness and mental health symptoms. Workplace loneliness was associated with psychiatric symptoms measured using the General Health Questionnaire (*r* = 0.35, *P* < 0.001; *r* = 0.57, *P* < 0.01) [40,44], the Patient Health Questionnaire (*r* = 0.29, *P* < 0.01) [43] and selected items from the Depression, Anxiety and Stress Scale (*r* = 0.23, gamma = 0.09, both *P* < 0.01) [41]. Workplace loneliness was also linked to job-related stress (*r* = 0.62, *P* < 0.01; *r* = 0.43, *P* < 0.05) [33,45,46], stress and weariness (both ρ = 0.44, *P* < 0.001) [47], and worse self-rated health (*r* = −0.27, *P* < 0.01) [39] and well-being (*r* = −0.32, *P* < 0.001) [48].

A smaller number of studies explored associations between workplace loneliness and workers’ personal characteristics. These studies found that loneliness was associated with lower extraversion [49] and rejection sensitivity [50], as well as lower core self-evaluation [51] and self-compassion [41].

When examining workplace loneliness measurement, we found that the majority of papers using quantitative methods used a validated instrument to assess workplace loneliness. Thirteen studies used an instrument designed to measure work-related loneliness; 11 studies adapted a general measure of loneliness to the workplace by adding phrases to contextualize items within the workplace, for example, ‘I feel left out [at work]’. Across the meta-analyses, associations were not different when a generic loneliness measure was adapted to the workplace or an instrument originally designed to measure workplace loneliness was used. However, there were too few studies and insufficient consistency in the loneliness measures to conduct subgroup analyses or meta-regression to examine the impact of measurement in-depth.

When examining the qualitative studies, our thematic synthesis generated three higher-order themes: (1) lonely jobs, (2) risk factors for workplace loneliness and (3) consequences of loneliness. Table 4 details the full thematic structure with illustrative quotes.

**Table 4.** Thematic structure with illustrative quotes

Higher-order theme	Sub-theme	Detail and illustrative quotes
Lonely jobs		Truck drivers: ‘I stay stressed all the time … Depressed, too … Feel sad, bad about being a truck driver. Lonely’. [52]
		Remote area medics: ‘Any person that tells you they don’t get lonely is bullshitting you’. [53]
		GPs: ‘It’s lonely work. I guess you have to get used to it in this work’. [63]
Risk factors for loneliness	Low frequency of social interactions at work	Working alone: ‘I get lonely quite often because I don’t have anyone to communicate with’. ([53])
		‘Well a lot can be said for loneliness because I mean, farming is a very singular sort of enterprise … and to be stuck out in the middle of nowhere feeding sheep 7 days a week … it can be very, very drawing and frustrating and very, very challenging’. [54]
		Working in isolation from colleagues: ‘Loneliness was a problem where I previously worked at X, I was alone there six years. One of my workmates asked why I came here, was it so I’d have workmates? But I said I’m really almost as much alone here as I was there, (laughter) even though I have a workmate in the next room … So, if there’s someone in the next room, it doesn’t necessarily mean one doesn’t feel alone (laughter) … And you don’t see anyone else unless you go visit them. So it’s lonely work’. [55]
	Barriers to meaningful, satisfying social connections	Lack of spontaneous, informal interactions: ‘In the office, we can chat with colleagues. Now [while working from home], we communicated only during meetings, but we did not talk about gossip or something interesting’. [56]
		Concern for professional image: ‘The reason it’s so lonely is we put those walls up … and nobody can know that I’m feeling, you know, concerned about my performance, that I’m insecure about this or that because football in a sense is ultimate meritocracy and such a manly thing that you just you always feel like you gotta be on, you know?’ [57]
		Social differences among co-workers: ‘There’s isolation monetarily within that locker room. They know if you either got it or don’t … And then race played a little bit of an issue in there … There’s still, socioeconomically, there’s a difference. How people were raised, there’s a difference. You know, if a guy’s working or if he’s tough guy or not. You know, so there’s a lot of things that can isolate guys’ [57]
		Telework—virtual interactions: ‘It’s just like you’re nurturing these relationships over an internet connection or over a phone and it’s not the same’ [65]
	Ways of working	Responsibility: ‘Nowadays this is quite lonely work, lonely and resembling an assembly line.... its good sides are independence and being close to the patients’ life and problems, but it also brings more responsibility, as no one else but you yourself will see the patients’ [55]
		*‘[Golf] is the loneliest game because it is really all up to you. It can just be a lonely game’.* [58]
		High workload: ‘Even though nurses were accustomed to meeting suicidal patients, they experienced that the loneliness increased when several patients were dealing with suicidal thoughts’. ([20]; analysis quote)
	Disconnected from society	‘The public will thank healthcare workers but then move away from you on a tram, or you get dirty looks when having a break in uniform. Very isolating’. [59]
Consequences of loneliness	Health and well-being	Mental health, distress: Within my first 3 weeks out, I actually broke down and cried one night when I was talking to my wife on the phone. I was that lonely … I had not talked to a single soul all day, and when I heard my wife’s voice, I just broke. [60]
		‘I’m always alone, man. I’m always alone. You know … it’s just that I know I can do more, but what do I do to make the money that I make? I’m sacrificing pretty much my sanity. My ability to talk to people. It is total isolation’. [52]
		Suicide: ‘I spoke to my 24-year-old son about my death … I’m no longer compassionate which has never been me. I really just want to stay home, I’m tired, lonely & sad and even writing this makes me feel guilty’. [59]
		Risky behaviour: ‘… the loneliness is the thing that bothers me and I think that’s what drives me to do a lot of stuff … It makes me seek out companionship in ways that I wouldn’t normally do [drug use and soliciting sex workers]’ [52]
	Changes in social behaviour	Withdrawal: ‘*You want to stay at home, you don’t want to see anybody … And I was like that with the telephone … I was at the stage where I didn’t want to talk to anyone on the phone, my wife had to talk to everyone. So you do, you just withdraw, you start to withdraw from society …’* [54]

		Seeking social interactions: *‘I have experienced loneliness … and sometimes I just go out to the shops or something just to have face to face interaction with somebody’.* [61]
		*[Loneliness] makes me seek out companionship in ways that I wouldn’t normally do* [52]
	Reduced confidence at work	‘The teleworkers emphasize the lack of social support available to talk things through which could produce other negative emotions such as feelings of insecurity and lack of confidence in their abilities’. ([61]; analysis quote)

Theme one, ‘lonely jobs’, reflects workers from a range of occupations’ reports that loneliness was a significant part of their working life. For some workers, loneliness was an intractable property of their job. Workers including doctors, farmers, truck drivers and professional athletes described their jobs as essentially and unchangeably lonely, suggesting feelings of powerlessness that may reflect workers’ limited ability to modify workplace social dynamics. A sample of remote area medics spoke candidly about the pervasiveness of loneliness in their work, with one participant stating that ‘any person [medic] that tells you they don’t get lonely is bulls***ing you’ [53].

Theme two, ‘risk factors for loneliness’, comprised a number of aspects of the work environment and working patterns that participants highlighted as generating loneliness. Participants first linked loneliness with spending time alone at work. This was often because their role was inherently solitary, such as for truck drivers [52] and farmers [54, 62]. Even for workers who spent significant periods of time with others during their workday, such as GPs, limited opportunities to interact with colleagues made their job ‘lonely work’ [55].

Workers also highlighted a number of barriers to developing satisfying relationships with colleagues, which elicited loneliness. Spontaneous, informal interactions were identified as important for building meaningful workplace relationships across multiple occupations. However, there was often little opportunity for these interactions, whether because workload impeded opportunities for interaction, meetings were discouraged [55], or online interactions focused only on work [56]. Virtual interactions were described as insufficient for building trusting relationships, both before and during the COVID-19 pandemic [63–65].

Responsibility and pressure at work were described as contributing to loneliness, particularly by healthcare workers. Nurses assessing suicide risk described feeling exposed in their responsibility to save lives [66], and GPs described both valuing independence and feeling isolated in making decisions with significant impact on patients [55]. This loneliness was exacerbated by a lack of feedback or consultation on their work and high workload [66].

Some workers linked negative societal attitudes towards their jobs with loneliness. Frontline healthcare workers described feeling ostracized, stigmatized and excluded by the public because of their occupation during the COVID-19 pandemic and linked this with loneliness [59]. A sample of Irish farmers echoed these feelings of being let down by society, stating that rural Ireland being ‘left [to] fend for itself’ contributed to their loneliness [62].

Participants also reported the impact of workplace loneliness, captured in the third theme ‘consequences of loneliness’. Workers from multiple occupations reported that workplace loneliness had negative consequences for their mental health and well-being, including depression [52,62,56,63,60], self-harm [54,59] and substance use [52]. Loneliness also contributed to reduced confidence and motivation at work [65,61], with participants describing feeling ‘stagnant’ [63].

Participants also stated that workplace loneliness led them to seek socialization outside work. While teleworkers described seeking socialization by going out after work [61], truck drivers sought connection in riskier ways, by soliciting sex workers or using drugs with others [53]. Conversely, farmers described withdrawing and avoiding others when lonely [54].

## Discussion

In this mixed-methods review of over two decades of research on workplace loneliness, we found consistent evidence that loneliness at work is related to workplace and well-being factors of interest to workers, employers and occupational health clinicians. Lonely workers had worse occupational functioning and well-being, pointing to potential implications for workers’ health and career progression, and possible costs for employers. Quantitative and qualitative evidence showed associations between work-related loneliness and the workplace social environment that are modifiable through intervention. This evidence emerged from a growing, but disparate literature on experiences of workplace loneliness. While we identified a substantial number of papers on the topic that covered a very wide range of correlates of workplace loneliness, the depth of the evidence was limited by the fact that few correlates were covered in two or more studies.

The reduced well-being and occupational functioning experienced by lonely workers highlights loneliness as a workplace health and productivity issue that deserves greater attention from employers and policy-makers. Associations with burnout, poor job performance, and lower job satisfaction echo research on loneliness in the general population that found that lonely individuals are more likely to take sick leave [67] or be unemployed [6] than their non-lonely peers. Links between workplace loneliness and reduced occupational functioning and burnout point to the implications of workplace loneliness for the well-being and career progression of workers, as well as economic costs for employers and society. However, as the evidence to date has almost exclusively relied on cross-sectional data, we cannot determine whether loneliness precipitates or is the result of poor well-being and occupational functioning. While work-related loneliness may contribute to burnout, low job satisfaction and reduced performance, the experience of performing poorly, being burnt-out, and feeling unsatisfied at work may conversely elicit loneliness. Longitudinal research is needed to better understand these associations and whether addressing workplace loneliness could improve workers’ well-being and workplace outcomes.

Associations with modifiable aspects of the workplace social environment also indicate the potential for targeting loneliness through interventions delivered in the workplace. While the direction of this association cannot be determined based on existing cross-sectional evidence, modifications to employer policies and training interventions that facilitate the development of supportive relationships at work may reduce loneliness among workers. In particular, we found substantial evidence for a link between workplace loneliness and the quality of workers’ relationships with their managers. The manager–worker relationship may be a particularly fruitful avenue for interventions targeting workplace loneliness because managers’ behaviour and attitudes can be effectively altered through training [68]. Indeed, managers’ ability to shape the working conditions of staff, knowledge of team and workplace issues, and capacity to model supportive professional relationships put them in an influential position to minimize or prevent the impact of other work-related risk factors for poor employee well-being. However, few interventions aiming to reduce loneliness in working-age adults have been delivered in the workplace, and those that have targeted workers’ skills and cognitions, rather than their work environment [69].

There was notable heterogeneity in the definition and conceptualization of work-related loneliness in the literature. Across the quantitative studies, workplace loneliness was operationalized using measures that contextualize participants’ feelings of loneliness within different parts of their work, including within their work in general, their organization, in relation to their colleagues, or their specific role. The workplace social environment is complex and comprises multiple, overlapping relationships and workers may feel dissatisfied with all or some of these relationships. The relative importance of loneliness across these different relationships is not known and has not been addressed in the research to date. Further, the distinctiveness of workplace loneliness from general loneliness is not clear, with most of the articles not adjusting for loneliness experienced outside of work. There is evidence that loneliness is trait-like for some people, such that some individuals are more likely to feel lonely across all environments [70], and it is possible that the loneliness measured in the studies included in our review is not specific to the workplace. Qualitative research exploring lived experiences of loneliness at work, combined with quantitative research that adjusts for loneliness in non-work domains, could improve understanding of the distinctiveness of workplace loneliness, its link with broader feelings of loneliness, and its particular risk factors and consequences for workers.

There were some gaps in the published literature. While a wide range of work-related correlates of loneliness were examined, the association between workplace loneliness and mental health problems was not widely examined. This reflects the predominance of research from business and management disciplines in the literature. Considering the established link between loneliness and poor mental health outcomes [7], mental health and occupational health researchers have a role to play in building the evidence base around experiences of work-related loneliness. Similarly, while a range of occupational groups were included in the studies, occupations at high risk of mental disorders, including first responders [71] and military personnel [72], as well as workers in insecure jobs in hospitality and the ‘gig economy’ were not included. Finally, most of the data used in the studies were collected before the COVID-19 pandemic. Social distancing measures and economic shifts have transformed working patterns and these results may not generalize to the current or future landscape of work.

The articles included in this review had some methodological limitations. Almost all of the quantitative studies were cross-sectional, and few adjusted for important confounders for loneliness such as depression. As such, the direction and robustness of the associations are untested and the risk factors and consequences of work-related loneliness remain unclear. The qualitative studies also varied in methodological quality. While some studies provided rich data on participants’ experiences of loneliness at work, others provided thin description and few quotes from participants to support interpretation.

There are also limitations to this review that should be considered. First, our search was limited to English-language publications, which may have resulted in some relevant studies being overlooked. However, as the workplace loneliness literature has emerged relatively recently, it is likely that English dominates the literature at this stage. Second, considerable heterogeneity was observed in the meta-analyses, warranting caution in the interpretation of pooled correlation coefficients despite the use of random-effects models. The small number of studies and diversity in measures and samples in the meta-analyses do not allow for the exploration of possible causes of the heterogeneity from sample occupation and loneliness measurement using meta-regression or subgroup analysis [19]. This level of heterogeneity is consistent with other meta-analyses examining the consequences of loneliness [8].

The findings from this study highlight the significance of workplace loneliness for employers, occupational health practitioners and researchers. Employers should be aware of the potential economic impact of reduced performance and burnout associated with workplace loneliness. Our findings also highlight the need for occupational health practitioners to be aware of loneliness alongside other aspects of workers’ well-being and consider its impact on their functioning at work. Evidence from the loneliness literature suggests that addressing negative social cognitions that are common among lonely individuals in a clinical therapeutic environment is effective in reducing general loneliness [20]. Adapting these interventions to the occupational health setting could be helpful for addressing loneliness in the workplace.

Our results also warrant a greater focus on the workplace context from loneliness researchers in mental health disciplines. In the context of the significant sickness absence burden of mental illness [9] and the established link between loneliness and mental health [7], there is a need for high-quality research on the impact of workplace loneliness on workers’ health and well-being. Further, while many loneliness interventions have been implemented in the community, the link between loneliness and modifiable aspects of the work environment provides the potential for interventions targeting loneliness to be delivered in the work environment. Finally, there is a need for research using longitudinal data and adjusting for confounders of loneliness to investigate the direction of these associations and identify risk factors and consequences of workplace loneliness.

This review has identified a growing literature on workplace loneliness. Emerging evidence indicates that loneliness is associated with reduced well-being and occupational functioning, which may have implications for workers, as well as significant costs for employers and the economy. Evidence also shows that loneliness is associated with modifiable aspects of the work environment, suggesting that the workplace may be a worthwhile avenue for future interventions targeting loneliness in the population. Further research using longitudinal data and adjusting for confounders of loneliness is needed to investigate the direction of these relationships and identify risk factors and consequences of workplace loneliness. There is a paucity of research examining associations between workplace loneliness and mental health. Mental health and occupational health researchers have a role to play in investigating experiences of loneliness at work and its impact on workers’ health and well-being.

## Supplementary Material

kqad138_suppl_Supplement_A_B_C

## Data Availability

This review was pre-registered on PROSPERO, CRD42021255553 https://bit.ly/3oG4vlj. All data, code, and research materials used in the meta-analyses are available at https://github.com/bridgetbryan/work-loneliness-review. For the purposes of open access, the author has applied a Creative Commons Attribution (CC BY) licence to any Accepted Author Manuscript version arising from this submission.

## References

[1] Department of Culture, Media & Sport. Employers and loneliness [Internet]. 2021 [cited 2023 June 28]. gov.uk/government/publications/employers-and-loneliness/employers-and-loneliness

[2] All Party Parliamentary Group on Loneliness and Connected Communities. Loneliness at Work [Internet]. 2023 [cited 2023 June 28]. redcross.org.uk/about-us/what-we-do/we-speak-up-for-change/loneliness-at-work

[3] New Economics Foundation. The Cost of Loneliness to UK Employers [Internet]. 2017 [cited 2023 June 28]. https://neweconomics.org/uploads/files/NEF_COST-OF-LONELINESS_DIGITAL-Final.pdf

[4] Perlman D , Peplau LA. Toward a social psychology of loneliness. Pers Relatsh 1981;3:31–56.

[5] Qualter P , Vanhalst J, Harris R et al. Loneliness across the life span. Perspect Psychol Sci 2015;10:250–264. 25910393 10.1177/1745691615568999

[6] Matthews T , Danese A, Caspi A et al. Lonely young adults in modern Britain: findings from an epidemiological cohort study. Psychol Med 2019;49:268–277. 29684289 10.1017/S0033291718000788 PMC6076992

[7] Park C , Majeed A, Gill H et al. The effect of loneliness on distinct health outcomes: a comprehensive review and meta-analysis. Psychiatry Res 2020;294:e113514. 10.1016/j.psychres.2020.113514 33130511

[8] Rico-Uribe LA , Caballero FF, Martín-María N, Cabello M, Ayuso-Mateos JL, Miret M. Association of loneliness with all-cause mortality: a meta-analysis. PLoS One 2018;13:e0190033. 29300743 10.1371/journal.pone.0190033 PMC5754055

[9] Henderson M , Harvey SB, Øverland S, Mykletun A, Hotopf M. Work and common psychiatric disorders. J R Soc Med 2011;104:198–207. 21558098 10.1258/jrsm.2011.100231 PMC3089873

[10] Koo B , Yu J, Chua BL, Lee S, Han H. Relationships among emotional and material rewards, job satisfaction, burnout, affective commitment, job performance, and turnover intention in the hotel industry. J Qual Assur Hosp Tour 2020;21:371–401.

[11] Wright S , Silard A. Unravelling the antecedents of loneliness in the workplace. Hum Relat 2021;74:1060–1081.

[12] Page MJ , McKenzie JE, Bossuyt PM et al. The PRISMA 2020 statement: an updated guideline for reporting systematic reviews. Int J Surg 2021;88:e105906. 10.1016/j.ijsu.2021.105906 33789826

[13] Ouzzani M , Hammady H, Fedorowicz Z, Elmagarmid A. Rayyan—a web and mobile app for systematic reviews. Syst Rev 2016;5:1–10. 27919275 10.1186/s13643-016-0384-4 PMC5139140

[14] Hong QN , Fàbregues S, Bartlett G et al. The Mixed Methods Appraisal Tool (MMAT) version 2018 for information professionals and researchers. Educ Inf 2018;34:285–291.

[15] StataCorp 2019 Stata Statistical Software: Release 16. College Station, TX: StataCorp LLC.

[16] Campbell M , McKenzie JE, Sowden A et al. Synthesis without meta-analysis (SWiM) in systematic reviews: reporting guideline. BMJ 2020;368:l6890. 31948937 10.1136/bmj.l6890 PMC7190266

[17] Thomas J , Harden A. Methods for the thematic synthesis of qualitative research in systematic reviews. BMC Med Res Methodol 2008;8:1–10. 18616818 10.1186/1471-2288-8-45 PMC2478656

[18] QSR International (2023) NVivo 14. Burlington: Lumivero.

[19] Deeks JJ , Higgins JPT, Altman DG; Cochrane Statistical Methods Group. Analysing data and undertaking meta‐analyses. In: Deeks JJ, Higgins JPT, Altman DG, and Cochrane Statistical Methods Group. Cochrane Handbook for Systematic Reviews of Interventions. Chichester: John Wiley & Sons. 2019. training.cochrane.org/handbook/current/chapter-10

[20] Masy CM , Chen HY, Hawkley LC, Cacioppo, JT. A meta-analysis of interventions to reduce loneliness. Pers Soc Psychol Rev 2011;15:219–266. 20716644 10.1177/1088868310377394 PMC3865701

[21] Cubitt S , Burt C. Leadership style, loneliness and occupational stress in New Zealand primary school principals. New Zealand J Educ Stud 2002;37:159–169.

[22] Fernet C , Torrès O, Austin S, St-Pierre J. The psychological costs of owning and managing an SME: linking job stressors, occupational loneliness, entrepreneurial orientation, and burnout. Burnout Res 2016;3:45–53.

[23] Amarat M , Akbolat M, Ünal O, Güneş Karakaya B. The mediating role of work alienation in the effect of workplace loneliness on nurses’ performance. J Nurs Manag 2018;27:553–559. 30171647 10.1111/jonm.12710

[24] Ozcelik H , Barsade SG. No employee an island: workplace loneliness and job performance. Acad Manag J 2018;61:2343–2366.

[25] Uslu O. ‘Being Alone is More Painful than Getting Hurt’: the moderating role of workplace loneliness in the association between workplace ostracism and job performance. *Cent Eur* Bus Rev 2021;10:19–38.

[26] Moens E , Baert S, Verhofstadt E, Van Ootegem L. Does loneliness lurk in temp work? exploring the associations between temporary employment, loneliness at work and job satisfaction. PLoS One 2021;16:e0250664. 33939743 10.1371/journal.pone.0250664 PMC8092765

[27] Spilker MA , Breaugh JA. Potential ways to predict and manage telecommuters’ feelings of professional isolation. J Vocat Behav 2021;131:e103646.

[28] Peng J , Chen Y, Xia Y, Ran Y. Workplace loneliness, leader-member exchange and creativity: the cross-level moderating role of leader compassion. Personal Individ Dif 2017;104:510–515.

[29] Arslan A , Yener S, Schermer JA. Predicting workplace loneliness in the nursing profession. J Nurs Manag 2020;28:710–717. 32106347 10.1111/jonm.12987

[30] Lam LW , Lau DC. Feeling lonely at work: investigating the consequences of unsatisfactory workplace relationships. Int J Human Resour Manag 2012;23:4265–4282.

[31] Bentein K , Garcia A, Guerrero S, Herrbach O. How does social isolation in a context of dirty work increase emotional exhaustion and inhibit work engagement? A process model. Personnel Rev 2017;46:1620–1634.

[32] Gabriel AS , Lanaj K, Jennings RE. Is one the loneliest number? A within-person examination of the adaptive and maladaptive consequences of leader loneliness at work. J Appl Psychol 2021;106:1517–1538. 33030923 10.1037/apl0000838

[33] Galanti T , Guidetti G, Mazzei E, Zappalà S, Toscano F. Work from home during the COVID-19 outbreak: the impact on employees’ remote work productivity, engagement, and stress. J Occup Environ Med 2021;63:e426–e432. 33883531 10.1097/JOM.0000000000002236 PMC8247534

[34] Golden TD , Veiga JF, Dino RN. The impact of professional isolation on teleworker job performance and turnover intentions: does time spent teleworking, interacting face-to-face, or having access to communication-enhancing technology matter? J Appl Psychol 2008;93:1412–1421. 19025257 10.1037/a0012722

[35] Orhan MA , Rijsman JB, van Dijk GM. Invisible, therefore isolated: comparative effects of team virtuality with task virtuality on workplace isolation and work outcomes. Eur J Work Organ Psychol 2016;32:109–122.

[36] Tutar H , Erdem AT. Examining the mediating role of organizational loneliness in the effect of organizational silence on the intention to quit. Uprav 2021;12:102–118.

[37] Zürcher A , Galliker S, Jacobshagen N, Lüscher Mathieu P, Eller A, Elfering A. Increased working from home in vocational counseling psychologists during COVID-19: associated change in productivity and job satisfaction. Front Psychol 2021;12:e750127. 10.3389/fpsyg.2021.750127 PMC8677673 34925154

[38] Graen GB , Uhl-Bien M. Relationship-based approach to leadership: Development of leader-member exchange (LMX) theory of leadership over 25 years: applying a multi-level multi-domain perspective. Leadersh Q 1995;6:219–247.

[39] Kuriakose V , Sreejesh S, Wilson PR, Anusree MR. The differential association of workplace conflicts on employee well-being: the moderating role of perceived social support at work. Int J Confl Manag 2019;30:680–705.

[40] Bentley TA , Teo ST, McLeod L, Tan F, Bosua R, Gloet M. The role of organisational support in teleworker wellbeing: a socio-technical systems approach. Appl Ergon 2016;52:207–215. 26360212 10.1016/j.apergo.2015.07.019

[41] Andel SA , Shen W, Arvan ML. Depending on your own kindness: the moderating role of self-compassion on the within-person consequences of work loneliness during the COVID-19 pandemic. J Occup Health Psychol 2021;26:276–290. 33734740 10.1037/ocp0000271

[42] Wang D , Liu H. Effects of job autonomy on workplace loneliness among knowledge workers. Chin Manag Stud 2020;15:182–195.

[43] Becker WJ , Belkin LY, Tuskey SE, Conroy SA. Surviving remotely: how job control and loneliness during a forced shift to remote work impacted employee work behaviours and well‐being. Hum Resour Manag 2022;61:449–464.

[44] Günther N , Hauff S, Gubernator P. The joint role of HRM and leadership for teleworker well-being: an analysis during the COVID-19 pandemic. German J Hum Resour Manag 2022;36:353–379.

[45] Guenzi P , Rangarajan D, Chaker NN, Sajtos L. It is all in good humor? Examining the impact of salesperson evaluations of leader humor on salesperson job satisfaction and job stress. J Person Selling Sales Manag 2019;39:352–369.

[46] Ulfert-Blank AS , Probst D, Scherer S, Green CS, Bowman ND, Greitemeyer T. Virtual work communication during a pandemic—the moderating effect of technology expertise on technology overload. Technol Mind Behav 2022;3:1–50.

[47] Hansen BG , Østerås O. Farmer welfare and animal welfare—exploring the relationship between farmer’s occupational well-being and stress, farm expansion and animal welfare. Prev Vet Med 2019;170:e104741. 10.1016/j.prevetmed.2019.104741 31421504

[48] D’Oliveira TC , Percisco L. Workplace isolation, loneliness and wellbeing at work: the mediating role of task interdependence and supportive behaviours. Appl Ergon 2023;106:103894. 36152448 10.1016/j.apergo.2022.103894

[49] Sîrbu AA , Dumbravă AC. Loneliness at work and job performance: the role of burnout and extraversion. Psihol Resur Um 2019;17:7–18.

[50] Khan HGA , Chughtai MS, Bashir A, Paracha UK. Rejection sensitivity and job performance: workplace loneliness as mediator and emotional culture of companionate love as moderator. Pakistan J Commer Soc Sci 2019;13:997–1016.

[51] Anand P , Mishra SK. Linking core self-evaluation and emotional exhaustion with workplace loneliness: does high LMX make the consequence worse? Int J Hum Resour Manag 2021;32:2124–2149.

[52] Shattell M , Apostolopoulos Y, Sönmez S, Griffin M. Occupational stressors and the mental health of truckers. Issues Ment Health Nurs 2010;31:561–568. 20701418 10.3109/01612840.2010.488783

[53] Whittaker-Howe S , Brown G, Williamson V, Greenberg N. The psychological health of remote area medics in Iraq. Occup Med (Lond) 2017;67:666–671. 29045714 10.1093/occmed/kqx138

[54] Perceval M , Kõlves K, Reddy P, De Leo D. Framer suicides: a qualitative study from Australia. Occup Med (Lond) 2017;67:383–388. 28633372 10.1093/occmed/kqx055

[55] Aira M , Mäntyselkä P, Vehviläinen A, Kumpusalo E. Occupational isolation among general practitioners in Finland. Occup Med (Lond) 2010;60:430–435. 20571099 10.1093/occmed/kqq082

[56] Wang B , Liu Y, Qian J, Parker SK. Achieving effective remote working during the COVID‐19 pandemic: a work design perspective. Appl Psychol 2021;70:16–59. 33230359 10.1111/apps.12290 PMC7675760

[57] McGraw SA , Deubert CR, Lynch HF, Nozzolillo A, Taylor L, Cohen IG. Life on an emotional roller coaster: NFL players and their family members’ perspectives on player mental health. J Clin Sport Psychol 2018;12:404–431.

[58] Fry J , Bloyce D. ‘Life in the Travelling Circus’: a study of loneliness, work stress, and money issues in touring professional golf. Sociol Sport J 2017;34:148–159.

[59] Bismark M , Smallwood N, Jain R, Willis K. Thoughts of suicide or self-harm among healthcare workers during the COVID-19 pandemic: qualitative analysis of open-ended survey responses. BJPsych Open 2022;8:e113. 35699151 10.1192/bjo.2022.509 PMC9203357

[60] Williams Jr DF , Thomas SP, Liao-Troth S. The truck driver experience: identifying psychological stressors from the voice of the driver. Transp J 2017;56:54–76.

[61] Mann S , Holdsworth L. The psychological impact of teleworking: stress, emotions and health. New Technol Work Employ 2003;18:196–211.

[62] Hammersley C , Richardson N, Meredith D, Carroll P, McNamara J. ‘That’s me i am the farmer of the land’: exploring identities, masculinities, and health among male farmers’ in Ireland. Am J Mens Health 2021;15:155798832110352–155798832110320. 10.1177/15579883211035241 PMC8383213 34414836

[63] Chaker NN , Nowlin EL, Walker D, Anaza NA. Alone on an island: a mixed-methods investigation of salesperson social isolation in general and in times of a pandemic. Ind Mark Manage 2021;96:268–286.

[64] Hersch E , Cohen KA, Saklecha A, Williams KD, Tan Y, Lattie EG. Remote-delivered services during COVID-19: a mixed-methods survey of college counselling centre clinicians. J Am Coll Health 2022;8:1–9. 10.1080/07448481.2022.2038178 PMC9848410 35259062

[65] McNaughton D , Rackensperger T, Dorn D, Wilson N. ‘Home is at work and work is at home’: telework and individuals who use augmentative and alternative communication. Work 2014;48:117–126. 24763351 10.3233/WOR-141860

[66] Jansson L , Graneheim UH. Nurses’ experiences of assessing suicide risk in specialised mental health outpatient care in rural areas. Issues Ment Health Nurs 2018;39:554–560. 29533690 10.1080/01612840.2018.1431823

[67] Mokros L , Świtaj P, Bieńkowski P, Święcicki L, Sienkiewicz-Jarosz H. Depression and loneliness may predict work inefficiency among professionally active adults. Int Arch Occup Environ Health 2022;95:1775–1783. 35503113 10.1007/s00420-022-01869-1 PMC9063248

[68] Gayed A , Milligan-Saville JS, Nicholas J et al. Effectiveness of training workplace managers to understand and support the mental health needs of employees: a systematic review and meta-analysis. Occup Environ Med 2018;75:462–470. 29563195 10.1136/oemed-2017-104789

[69] Cacioppo JT , Adler AB, Lester PB et al. Building social resilience in soldiers: a double dissociative randomized controlled study. J Pers Soc Psychol 2015;109:90–105. 26098588 10.1037/pspi0000022

[70] Mund M , Freuding MM, Möbius K, Horn N, Neyer FJ. The stability and change of loneliness across the life span: a meta-analysis of longitudinal studies. Pers Soc Psychol Rev 2020;24:24–52. 31179872 10.1177/1088868319850738 PMC6943963

[71] Petrie K , Milligan-Saville J, Gayed A et al. Prevalence of PTSD and common mental disorders amongst ambulance personnel: a systematic review and meta-analysis. Soc Psychiatry Psychiatr Epidemiol 2018;53:897–909. 29869691 10.1007/s00127-018-1539-5

[72] Goodwin L , Wessely S, Hotopf M et al. Are common mental disorders more prevalent in the UK serving military compared to the general working population? Psychol Med 2015;45:1881–1891. 25602942 10.1017/S0033291714002980

