# Supplementary material for: Loneliness in the workplace: a mixed-method systematic review and meta-analysis
Source: Occup Med (Lond). 2024 Jan 29;73(9):557–67. doi: 10.1093/occmed/kqad138 (PMC10824263; doi:10.1093/occmed/kqad138)
Supplement: kqad138_suppl_Supplement_A_B_C [file kqad138_suppl_supplement_a_b_c.docx]

**Loneliness in the workplace:**

**A mixed-method systematic review and meta-analysis**

# Supplement A: Search strategy

While this review focuses on loneliness, which is distinct from social isolation [6], as some research has used the term ‘isolation’ to describe work-related loneliness [16-17], we included terms related to isolation to ensure comprehensiveness.

**Table S1. Search terms for each database**

| **Database** | **Loneliness AND** | **Workplace AND** | **Mental health OR** | **Physical health OR** | **Work factors OR** |
| --- | --- | --- | --- | --- | --- |
| **PsycINFO** | lonel*.ab,ti | worker.ab,ti | mental health.ab,ti | morbidity.ab,ti | job satis*.ab,ti |
|  | social isolat*.ab,ti | workplace.ab,ti | wellbeing.ab,ti | illness.ab,ti | work satis*.ab,ti |
|  |  | employe*.ab,ti | well being.ab,ti | disease.ab,ti | job performance.ab,ti |
|  | exp Loneliness/ | occupat*.ab,ti | well-being.ab,ti | injury.ab,ti | work performance.ab,ti |
|  | exp Social Isolation/ | job.ab,ti | depress*.ab,ti | pain.ab,ti | employee performance.ab,ti |
|  |  |  | anxi*.ab,ti | smoking.ab,ti | work engagement.ab,ti |
|  |  | exp Occupational Health/ | mood.ab,ti | tobacco use.ab,ti | job engagement.ab,ti |
|  |  | exp Job Characteristics | psychological distress.ab,ti | drug use.ab,ti | intent to stay.ab,ti |
|  |  |  | resilien*.ab,ti | substance use.ab,ti | absenteeism.ab,ti |
|  |  |  | burnout.ab,ti | alcohol use.ab,ti | presenteeism.ab,ti |
|  |  |  | life satisfaction.ab,ti | exercise.ab,ti |  |
|  |  |  | happiness.ab,ti | physical activity.ab,ti | exp Job Satisfaction/ |
|  |  |  | quality of life.ab,ti | diet.ab,ti | exp Job Performance/ |
|  |  |  |  | weight.ab,ti | exp Occupational Stress/ |
|  |  |  | exp Mental Disorders/ | obesity.ab,ti | exp Employee Absenteeism/ |
|  |  |  | exp Mental Health/ |  |  |
|  |  |  | exp Psychological Stress/ | exp Work Related Illnesses/ |  |
|  |  |  |  | exp Health Behavior/ |  |
|  |  |  |  | exp Tobacco Smoking/ |  |
|  |  |  |  | exp Alcohol Drinking Patterns/ |  |
|  |  |  |  | exp Exercise/ |  |
|  |  |  |  | exp Obesity/ |  |
| **Medline** | lonel*.ab,ti | worker.ab,ti | mental health.ab,ti | morbidity.ab,ti | job satis*.ab,ti |
|  | social isolat*.ab,ti | workplace.ab,ti | wellbeing.ab,ti | illness.ab,ti | work satis*.ab,ti |
|  |  | employe*.ab,ti | well being.ab,ti | disease.ab,ti | job performance.ab,ti |
|  | exp Loneliness/ | occupat*.ab,ti | well-being.ab,ti | injury.ab,ti | work performance.ab,ti |
|  | exp Social Isolation/ | job.ab,ti | depress*.ab,ti | pain.ab,ti | employee performance.ab,ti |
|  |  |  | anxi*.ab,ti | smoking.ab,ti | work engagement.ab,ti |
|  |  | exp Workplace/ | mood.ab,ti | tobacco use.ab,ti | job engagement.ab,ti |
|  |  | exp Occupational Helath/ | psychological distress.ab,ti | drug use.ab,ti | intent to stay.ab,ti |
|  |  |  | resilien*.ab,ti | substance use.ab,ti | absenteeism.ab,ti |
|  |  |  | burnout.ab,ti | alcohol use.ab,ti | presenteeism.ab,ti |
|  |  |  | life satisfaction.ab,ti | exercise.ab,ti |  |
|  |  |  | happiness.ab,ti | physical activity.ab,ti | exp Job Satisfaction/ |
|  |  |  | quality of life.ab,ti | diet.ab,ti | exp Work Performance/ |
|  |  |  |  | weight.ab,ti | exp Work Engagement/ |
|  |  |  | exp Mental Health/ | obesity.ab,ti |  |
|  |  |  | exp Mental Disorders/ |  |  |
|  |  |  | exp Stress, Psychological/ | exp Occupational Disease/ |  |
|  |  |  |  | exp Health Behaviour/ |  |
|  |  |  |  | exp Tobacco Smoking/ |  |
|  |  |  |  | exp Alcohol Drinking/ |  |
|  |  |  |  | exp Exercise/ |  |
|  |  |  |  | exp Obesity/ |  |
| **Embase** | lonel*.ab,ti | worker.ab,ti | mental health.ab,ti | morbidity.ab,ti | job satis*.ab,ti |
|  | social isolat*.ab,ti | workplace.ab,ti | wellbeing.ab,ti | illness.ab,ti | work satis*.ab,ti |
|  |  | employe*.ab,ti | well being.ab,ti | disease.ab,ti | job performance.ab,ti |
|  | exp Loneliness/ | occupat*.ab,ti | well-being.ab,ti | injury.ab,ti | work performance.ab,ti |
|  | exp Social Isolation/ | job.ab,ti | depress*.ab,ti | pain.ab,ti | employee performance.ab,ti |
|  |  |  | anxi*.ab,ti | smoking.ab,ti | work engagement.ab,ti |
|  |  | exp Work Environment/ | mood.ab,ti | tobacco use.ab,ti | job engagement.ab,ti |
|  |  | exp Occupational Health/ | psychological distress.ab,ti | drug use.ab,ti | intent to stay.ab,ti |
|  |  |  | resilien*.ab,ti | substance use.ab,ti | absenteeism.ab,ti |
|  |  |  | burnout.ab,ti | alcohol use.ab,ti | presenteeism.ab,ti |
|  |  |  | life satisfaction.ab,ti | exercise.ab,ti |  |
|  |  |  | happiness.ab,ti | physical activity.ab,ti | exp Absenteeism/ |
|  |  |  | quality of life.ab,ti | diet.ab,ti | exp Job performance/ |
|  |  |  |  | weight.ab,ti | exp Job satisfaction/ |
|  |  |  | exp Mental Health/ | obesity.ab,ti | exp Work engagement/ |
|  |  |  | exp Mental Disease/ |  |  |
|  |  |  | exp Mental Stress/ | exp Occupational Disease/ |  |
|  |  |  |  | exp Health Behaviour/ |  |
|  |  |  |  | exp Smoking/ |  |
|  |  |  |  | exp Drinking Behaviour/ |  |
|  |  |  |  | exp Exercise/ |  |
|  |  |  |  | exp Obesity/ |  |
| **PubMed** | lonel*[Title/Abstract] | worker[Title/Abstract] | mental health[Title/Abstract] | morbidity[Title/Abstract] | job satis*[Title/Abstract] |
|  | social isolat*[Title/Abstract] | workplace[Title/Abstract] | depress*[Title/Abstract] | illness[Title/Abstract] | work satis*[Title/Abstract] |
|  | loneliness[MeSH Terms] | employe*[Title/Abstract] | anxi*[Title/Abstract] | disease[Title/Abstract] | job performance[Title/Abstract] |
|  | social isolation[MeSH Terms] | occupat*[Title/Abstract] | mood[Title/Abstract] | injury[Title/Abstract] | work performance[Title/Abstract] |
|  |  | job[Title/Abstract] | psychological distress[Title/Abstract] | pain[Title/Abstract] | employee performance[Title/Abstract] |
|  |  | workplace[MeSH Terms] | wellbeing[Title/Abstract] | smoking[Title/Abstract] | work engagement[Title/Abstract] |
|  |  | occupational health[MeSH Terms] | well being[Title/Abstract] | tobacco use[Title/Abstract] | job engagement[Title/Abstract] |
|  |  |  | happiness[Title/Abstract] | drug use[Title/Abstract] | intent to stay[Title/Abstract] |
|  |  |  | life satisfaction[Title/Abstract] | substance use[Title/Abstract] | absenteeism[Title/Abstract] |
|  |  |  | quality of life[Title/Abstract] | alcohol use[Title/Abstract] | presenteeism[Title/Abstract]) |
|  |  |  | resilien*[Title/Abstract] | exercise[Title/Abstract] | job satisfaction[MeSH Terms] |
|  |  |  | burnout[Title/Abstract] | physical activity[Title/Abstract] | absenteeism[MeSH Terms] |
|  |  |  | mental health[MeSH Terms] | diet[Title/Abstract] |  |
|  |  |  | stress, psychological[MeSH Terms] | weight[Title/Abstract] |  |
|  |  |  |  | obesity[Title/Abstract]) |  |
|  |  |  |  | health behavior[MeSH Terms] |  |
|  |  |  |  | tobacco smoking[MeSH Terms] |  |
|  |  |  |  | alcohol drinking[MeSH Terms] |  |
|  |  |  |  | exercise[MeSH Terms] |  |
|  |  |  |  | occupational diseases[MeSH Terms] |  |
| **EBSCO Business Source Complete** | TI,AB lonel* | TI,AB worker | TI,AB mental health | TI,AB morbidity | TI,AB job satisfaction |
|  | TI,AB social isolat* | TI,AB workplace | TI,AB depress* | TI,AB illness | TI,AB work satisfaction |
|  |  | TI,AB employe* | TI,AB anxi* | TI,AB disease | TI,AB employee satisfaction |
|  |  | TI,AB occupat* | TI,AB mood | TI,AB injury | TI,AB job performant |
|  |  | TI,AB job | TI,AB psychological distress | TI,AB pain | TI,AB work performance |
|  | SU loneliness |  | TI,AB wellbeing | TI,AB smoking | TI,AB employee perfromance |
|  | SU social isolation |  | TI,AB well being | TI,AB tobacco use | TI,AB job engagement |
|  |  |  | TI,AB resilien* | TI,AB drug use | TI,AB work engagement |
|  |  |  | TI,AB burnout | TI,AB substance use | TI,AB intent to stay |
|  |  |  | TI,AB burn-out | TI,AB alcohol use | TI,AB absenteeism |
|  |  |  |  | TI,AB exercise | TI,AB presenteeism |
|  |  | SU workplace | SU mental health | TI,AB physical activity |  |
|  |  | SU occupational health | SU psychological stress | TI,AB diet | SU job satisfaction |
|  |  | SU work environment |  | TI,AB weight | SU work performance |
|  |  |  |  | TI,AB obesity | SU work engagement |
|  |  |  |  |  |  |
|  |  |  |  | SU health behaviour |  |
|  |  |  |  | SU health behaviour |  |
|  |  |  |  | SU occupational health |  |
|  |  |  |  | SU alcohol consumption |  |
|  |  |  |  | SU exercise or physical activity |  |
|  |  |  |  | SU obesity |  |

# Supplement B: Evidence synthesis methods

We synthesised the evidence using a parallel-results convergent approach, where qualitative and quantitative articles were analysed separately before being integrated in the final synthesis [1].

**Meta-analyses**

Random effects meta-analyses were performed in Stata 17 using the *meta summarize* command. Studies’ samples and construct measurement varied, so we used random effects models to calculate conservative estimates of pooled effect size. Correlation coefficients were transformed using Fisher’s z transformation [2] for meta-analysis. Pooled effect sizes were then converted back to, and are reported as, Pearson’s correlation coefficients with 95% confidence intervals. As few studies were eligible for meta-analysis, it was not appropriate to investigate heterogeneity using meta-regression or sub-group analysis.

**Thematic synthesis of qualitative data**

We synthesised qualitative data using Thomas and Harden’s thematic synthesis approach [3], informed by thematic analysis [4]. Quotes and themes relating to workplace loneliness were extracted and imported into NVivo software and were read line-by-line and coded for analytically salient features by two researchers (BTB and GA) independently. These codes were then reviewed and analysed to construct a descriptive theme structure. The themes were discussed in detail by the two coders where the content, meaning, and name of each theme was reviewed and refined.

1. Hong QN, Pluye P, Bujold M, Wassef M. Convergent and sequential synthesis designs: implications for conducting and reporting systematic reviews of qualitative and quantitative evidence. *Syst. Rev.* 2017;6:1-14.
2. Welz T, Doebler P, Pauly M. Fisher transformation-based confidence intervals of correlations in fixed- and random-effects meta-analysis. *Br J Math Stat Psychol* 2022;75:1-22.
3. Thomas J, Harden A. Methods for the thematic synthesis of qualitative research in systematic reviews. *BMC Medical Res. Methodol.* 2008;8:1-10.
4. Braun V, Clarke V. Using thematic analysis in psychology. *Qual. Res. Psychol.* 2006;3:77-101.

# Supplement C. Full list of articles included in the systematic review

Aira, M., Mäntyselkä, P., Vehviläinen, A., & Kumpusalo, E. (2010). Occupational isolation among general practitioners in Finland. *Occupational Medicine*, *60*(6), 430-435. https://doi.org/10.1093/occmed/kqq082

Amarat, M., Akbolat, M., Ünal, Ö., & Güneş Karakaya, B. (2019). The mediating role of work alienation in the effect of workplace loneliness on nurses’ performance. *Journal of Nursing Management*, *27*(3), 553-559. https://doi.org/10.1111/jonm.12710

Anand, P. & Mishra, S. K. (2021). Linking core self-evaluation and emotional exhaustion with workplace loneliness: does high LMX make the consequence worse? *The International Journal of Human Resource Management*, *32*(10), 2124-2149. https://doi.org/10.1080/09585192.2019.1570308

Andel, S. A., Shen, W., & Arvan, M. L. (2021). Depending on your own kindness: The moderating role of self-compassion on the within-person consequences of work loneliness during the COVID-19 pandemic. *Journal of Occupational Health Psychology, 26*(4), 276-290. https://doi.org/10.1037/ocp0000271

Arslan, A., Yener, S., & Schermer, J. A. (2020). Predicting workplace loneliness in the nursing profession. *Journal of Nursing Management*, *28*(3), 710-717. https://doi.org/10.1111/jonm.12987

Becker, W. J., Belkin, L. Y., Tuskey, S. E., & Conroy, S. A. (2022). Surviving remotely: How job control and loneliness during a forced shift to remote work impacted employee work behaviours and well‐being. *Human Resource Management, 61*(4), 449-464. https://doi.org/10.1002/hrm.22102

Bentein, K., Garcia, A., Guerrero, S., & Herrbach, O. (2017). How does social isolation in a context of dirty work increase emotional exhaustion and inhibit work engagement? A process model. *Personnel Review, 46*(8), 1620-1634. https://doi.org/10.1108/PR-09-2016-0227

Bentley, T. A., Teo, S. T., McLeod, L., Tan, F., Bosua, R., & Gloet, M. (2016). The role of organisational support in teleworker wellbeing: A socio-technical systems approach. *Applied Ergonomics*, *52*, 207-215. https://doi.org/10.1016/j.apergo.2015.07.019

Bismark, M., Smallwood, N., Jain, R., & Willis, K. (2022). Thoughts of suicide or self-harm among healthcare workers during the COVID-19 pandemic: qualitative analysis of open-ended survey responses. *BJPsych Open*, *8*(4). https://doi.org/10.1192%2Fbjo.2022.509

Chaker, N. N., Nowlin, E. L., Walker, D., & Anaza, N. A. (2021). Alone on an island: A mixed-methods investigation of salesperson social isolation in general and in times of a pandemic. *Industrial Marketing Management*, *96*, 268-286. https://doi.org/10.1016/j.indmarman.2021.05.009

Cubitt, S. & Burt, C. (2002). Leadership Style, Loneliness and Occupational Stress in New Zealand Primary School Principals. *New Zealand Journal of Educational Studies*, *37*(2), 159-169.

D’Oliveira, T. C., & Percisco, L. (2023). Workplace Isolation, Loneliness and Wellbeing at Work: The Mediating Role of Task Interdependence and Supportive Behaviours. *Applied Ergonomics*, *106*. https://doi.org/10.1016/j.apergo.2022.103894

Fernet, C., Torrès, O., Austin, S., & St-Pierre, J. (2016). The psychological costs of owning and managing an SME: Linking job stressors, occupational loneliness, entrepreneurial orientation, and burnout. *Burnout Research*, *3*(2), 45-53. https://doi.org/10.1016/j.burn.2016.03.002

Fry, J., & Bloyce, D. (2017). ‘Life in the Travelling Circus’: A Study of Loneliness, Work Stress, and Money Issues in Touring Professional Golf. *Sociology of Sport Journal*, *34*(2), 148-159. https://doi.org/10.1123/ssj.2017-0002

Gabriel, A. S., Lanaj, K., & Jennings, R. E. (2021). Is one the loneliest number? A within-person examination of the adaptive and maladaptive consequences of leader loneliness at work. *Journal of Applied Psychology, 106*(10), 1517–1538. https://doi.org/10.1037/apl0000838

Galanti, T., Guidetti, G., Mazzei, E., Zappalà, S., & Toscano, F. (2021). Work From Home During the COVID-19 Outbreak: The Impact on Employees’ Remote Work Productivity, Engagement, and Stress. *Journal of Occupational and Environmental Medicine*, *63*(7), 426-432. https://doi.org/10.1097%2FJOM.0000000000002236

Gascon, H. (2009). Self-esteem, loneliness, satisfaction of adults with intellectual disabilities (ID) in sheltered or regular workplace. *British Journal of Developmental Disabilities*, *55*(109), 145-155. https://doi.org/10.1179/096979509799103124

Golden, T. D., Veiga, J. F., & Dino, R. N. (2008). The impact of professional isolation on teleworker job performance and turnover intentions: Does time spent teleworking, interacting face-to-face, or having access to communication-enhancing technology matter? *Journal of Applied Psychology, 93*(6), 1412–1421. https://doi.org/10.1037/a0012722

Guenzi, P., Rangarajan, D., Chaker, N. N., & Sajtos, L. (2019). It is all in good humor? Examining the impact of salesperson evaluations of leader humor on salesperson job satisfaction and job stress. *Journal of Personal Selling & Sales Management*, *39*(4), 352-369. https://doi.org/10.1080/08853134.2019.1598267

Günther, N., Hauff, S., & Gubernator, P. (2022). The joint role of HRM and leadership for teleworker well-being: An analysis during the COVID-19 pandemic. *German Journal of Human Resource Management*, *36*(3), 353-379. https://doi.org/10.1177/23970022221083694

Hammersley, C., Richardson, N., Meredith, D., Carroll, P., & McNamara, J. (2021). “That’s Me I am the Farmer of the Land”: Exploring Identities, Masculinities, and Health Among Male Farmers’ in Ireland. *American Journal of Men's Health*, *15*(4), 1-20. https://doi.org/10.1177/15579883211035241

Hansen, B. G., & Østerås, O. (2019). Farmer welfare and animal welfare- Exploring the relationship between farmer’s occupational well-being and stress, farm expansion and animal welfare. *Preventive Veterinary Medicine*, *170,* https://doi.org/10.1016/j.prevetmed.2019.104741

Hersch, E., Cohen, K. A., Saklecha, A., Williams, K. D., Tan, Y., & Lattie, E. G. (2022). Remote-delivered services during COVID-19: A mixed-methods survey of college counselling centre clinicians. *Journal of American College Health.* Advance online publication. https://doi.org/10.1080/07448481.2022.2038178

Jansson, L., & Graneheim, U. H. (2018). Nurses' Experiences of Assessing Suicide Risk in Specialised Mental Health Outpatient Care in Rural Areas. *Issues in Mental Health Nursing*, *39*(7), 554-560. https://doi.org/10.1080/01612840.2018.1431823

Keskin, A. Y., & Şentürk, S. (2022). Being A Hemodialysis Nurse During the COVID-19 Pandemic: A Phenomenological Approach. *OMEGA - Journal of Death and Dying*, *0*(0), 1-19. https://doi.org/10.1177%2F00302228221090755

Khan, H. G. A., Chughtai, M. S., Bashir, A., & Paracha, U. K. (2019). Rejection Sensitivity and Job Performance: Workplace Loneliness as Mediator and Emotional Culture of Companionate Love as Moderator. *Pakistan Journal of Commerce and Social Sciences*, *13*(4), 997-1016.

Kuriakose, V., Sreejesh, S., Wilson, P. R., & Anusree, M. R. (2019). The differential association of workplace conflicts on employee well-being: The moderating role of perceived social support at work. *International Journal of Conflict Management*, *30*(5), 680-705. https://doi.org/10.1108/IJCMA-05-2018-0063

Lam, L. W., & Lau, D. C. (2012). Feeling lonely at work: investigating the consequences of unsatisfactory workplace relationships. *The International Journal of Human Resource Management*, *23*(20), 4265-4282. https://doi.org/10.1080/09585192.2012.665070

Mann, S., & Holdsworth, L. (2003). The psychological impact of teleworking: stress, emotions and health. *New Technology, Work and Employment*, *18*(3), 196-211. https://doi.org/10.1111/1468-005X.00121

McGraw, S. A., Deubert, C. R., Lynch, H. F., Nozzolillo, A., Taylor, L., & Cohen, I. G. (2018). Life on an Emotional Roller Coaster: NFL Players and Their Family Members’ Perspectives on Player Mental Health. *Journal of Clinical Sport Psychology*, *12*(3), 404-431. https://doi.org/10.1123/jcsp.2017-0051

McNaughton, D., Rackensperger, T., Dorn, D., & Wilson, N. (2014). " Home is at work and work is at home": Telework and individuals who use augmentative and alternative communication. *Work*, *48*(1), 117-126. https://doi.org/10.3233/wor-141860

Moens, E., Baert, S., Verhofstadt, E., & Van Ootegem, L. (2021). Does loneliness lurk in temp work? Exploring the associations between temporary employment, loneliness at work and job satisfaction. *PloS One*, *16*(5), Article e0250664. https://doi.org/10.1371/journal.pone.0250664

Orhan, M. A., Rijsman, J. B., & van Dijk, G. M. (2016). Invisible, therefore isolated: Comparative effects of team virtuality with task virtuality on workplace isolation and work outcomes. *Journal of Work and Organizational Psychology*, *32*(2), 109-122. https://doi.org/10.1016/j.rpto.2016.02.002

Ozcelik, H., & Barsade, S. G. (2018). No employee an island: Workplace loneliness and job performance. *Academy of Management Journal*, *61*(6), 2343-2366. https://doi.org/10.5465/amj.2015.1066

Peng, J., Chen, Y., Xia, Y. & Ran, Y. (2017). Workplace loneliness, leader-member exchange and creativity: The cross-level moderating role of leader compassion. *Personality and Individual Differences, 104*(1), 510-515. https://doi.org/10.1016/j.paid.2016.09.020

Perceval, M., Kõlves, K., Reddy, P., De Leo, D., (2017). Framer suicides: a qualitative study from Australia. *Occupational Medicine*, *67*(5), 383–388. https://doi.org/10.1093/occmed/kqx055

Satilmis, A., Oznacar, B., Uzunboylu, H., & Yılmaz, E. (2018). The life satisfaction of teachers at work place, research of structural equation modelling regarding general and organized cynicism. *Quality & Quantity: International Journal of Methodology*, *52*(1), 1-10. https://doi.org/10.1007/s11135-017-0578-4

Shattell, M., Apostolopoulos, Y., Sönmez, S., & Griffin, M. (2010). Occupational Stressors and the Mental Health of Truckers. *Issues in Mental Health Nursing*, *31*(9), 561-568. https://doi.org/10.3109/01612840.2010.488783

Silard, A., & Wright, S. (2022). Distinctly lonely: how loneliness at work varies by status in organizations. *Management Research Review*, *45*(7), 913-928. http://doi.org/10.1108/MRR-05-2021-0379

Silman, F., & Dogan, T. (2013). Social Intelligence as a Predictor of Loneliness in the Workplace. *The Spanish Journal of Psychology,* *16*, e36. https://doi.org/10.1017/sjp.2013.21

Sîrbu, A. A., & Dumbravă, A. C. (2019). Loneliness at Work and Job Performance: The Role of Burnout and Extraversion. *Psihologia Resurselor Umane*, *17*(1), 7-18. http://dx.doi.org/10.24837/pru.2019.1.491

Spilker, M. A., & Breaugh, J. A. (2021). Potential ways to predict and manage telecommuters' feelings of professional isolation. *Journal of Vocational Behavior*, *131*, Article e103646. https://doi.org/10.1016/j.jvb.2021.103646

Tutar, H., & Erdem, A. T., (2021). Examining the mediating role of organizational loneliness in the effect of organizational silence on the intention to quit. *Upravlenets (The Manager)*, *12*(2), 102-118. https://doi.org/10.29141/2218-5003-2021-12-2-8

Ulfert-Blank, A. S., Probst, D., Scherer, S., Green, C. S., Bowman, N. D., & Greitemeyer, T. (2022). Virtual Work Communication During a Pandemic—The Moderating Effect of Technology Expertise on Technology Overload. *Technology, Mind, and Behavior*, *3*(2). https://doi.org/10.1037/tmb0000071

Uslu, O. (2021). “Being Alone is More Painful than Getting Hurt”: The Moderating Role of Workplace Loneliness in the Association Between Workplace Ostracism and Job Performance. *Central European Business Review*, *10*(1), 19-38. https://doi.org/10.18267/j.cebr.257

Wang, B., Liu, Y., Qian, J., & Parker, S. K. (2021). Achieving Effective Remote Working During the COVID‐19 Pandemic: A Work Design Perspective. *Applied Psychology*, *70*(1), 16-59. https://doi.org/10.1111/apps.12290

Whittaker-Howe, S., Brown, G., Williamson, V., & Greenberg, N. (2017). The psychological health of remote area medics in Iraq. *Occupational Medicine*, *67*(9), 666-671. https://doi.org/10.1093/occmed/kqx138

Williams Jr, D. F., Thomas, S. P., & Liao-Troth, S. (2017). The Truck Driver Experience: Identifying Psychological Stressors from the Voice of the Driver. *Transportation Journal*, *56*(1), 54-76. https://doi.org/10.5325/transportationj.56.1.0054

Zürcher, A., Galliker, S., Jacobshagen, N., Lüscher Mathieu, P., Eller, A., & Elfering, A. (2021). Increased Working From Home in Vocational Counseling Psychologists During COVID-19: Associated Change in Productivity and Job Satisfaction. *Frontiers in Psychology*, *12*, Article e750127. https://doi.org/10.3389/fpsyg.2021.750127
